# Single-position oblique lumbar interbody fusion with navigation: improved efficiency and screw accuracy compared to dual-position with fluoroscopy

**DOI:** 10.1038/s41598-024-67007-8

**Published:** 2024-07-23

**Authors:** Hangeul Park, Hui Son, Jun-Hoe Kim, Sum Kim, Young-Rak Kim, Chang-Hyun Lee, Chun Kee Chung, Chi Heon Kim

**Affiliations:** 1 Department of Neurosurgery, Seoul National University Hospital, Seoul, Republic of Korea; 2 College of Medicine, Seoul National University, Seoul, Republic of Korea; 3 Department of Neurosurgery, Kangdong Sacred Heart Hospital, Hallym University College of Medicine, Seoul, Republic of Korea; 4 Department of Neurosurgery, Armed Forces Yangju Hospital, Yangju, Republic of Korea; 5 Department of Neurosurgery, Seoul National University College of Medicine, Seoul, Republic of Korea; 6 Neuroscience Research Institute, Seoul National University Medical Research Center, Seoul, Republic of Korea; 7 Department of Medical Device Development, Seoul National University College of Medicine, Seoul, Republic of Korea

**Keywords:** Neurological disorders, Neuropathic pain, Chronic pain

## Abstract

Dual-position oblique lumbar interbody fusion with fluoroscopy (D-OLIF) requires repositioning the patient to a prone position for pedicle screw insertion. Recently, single-position surgery with navigation has been introduced. However, there are concerns regarding pedicle screw accuracy and achieving appropriate sagittal balance in single-position OLIF with navigation (S-OLIF). The purpose of this study is to evaluate the clinical and radiological outcomes of S-OLIF compared to D-OLIF. A retrospective analysis was conducted on 102 patients who underwent single-level OLIF at a single institution. The patients were divided into two groups: 55 in the S-OLIF group and 47 in the D-OLIF group. The numeric rating scale for back and leg, Oswestry disability index, and walking distance improvements showed no significant difference. However, the EuroQol 5-dimension 5-level index showed higher improvement in the S-OLIF (P = 0.029). The segmental lordosis, lumbar lordosis, and C7 sagittal vertical axis showed no significant difference. S-OLIF had significantly fewer cases of pedicle screw malposition (P = 0.045). Additionally, the surgery time was shorter in the S-OLIF (P = 0.002). In conclusion, S-OLIF exhibited clinical and radiological outcomes comparable to D-OLIF, with the added advantages of reduced surgery time and enhanced accuracy in pedicle screw placement.

## Introduction

Lumbar interbody fusion has become a widely utilized surgical technique for treating various lumbar spine conditions, such as degenerative disc disease, spondylolisthesis (SPL), and spinal stenosis^1,2^. Among the minimally invasive approaches, oblique lumbar interbody fusion (OLIF) offers several advantages, such as reduced muscle trauma, shorter hospital stays, and faster recovery compared to the posterior approach^3,4^. Furthermore, it presents a lower risk of vascular, bowel, and reproductive injuries in comparison to the anterior approach^5,6^. In the dural-position OLIF with fluoroscopy (D-OLIF), the patients are repositioned from a lateral decubitus to a prone position for the insertion of pedicle screws^7,8^. However, the recent advancements in intraoperative navigation systems have allowed the placement of pedicle screws in the lateral position without requiring a position change^7,9^. Single-position OLIF with navigation (S-OLIF) streamlines the surgical workflow, reduces operative time, and potentially lowers the risk of complications associated with patient repositioning^10^. Additionally, S-OLIF may result in less intraoperative blood loss and shorter hospital stays, contributing to improved patient outcomes and faster postoperative recovery^7,9^. Despite these benefits, concerns remain about the lateral position for pedicle screw insertion, as surgeons may be less familiar with this approach, potentially leading to the risk of screw misplacement^11^. Moreover, compared to the prone position, the lateral decubitus position may not provide sufficient segmental lordosis (SL) and lumbar lordosis (LL), which could be a limitation in improving sagittal imbalance^12^. The objective of this study is to evaluate the clinical and radiological outcomes of S-OLIF compared to D-OLIF in patients with lumbar spine disorders.

## Methods

### Study population

A retrospective analysis of prospectively collected data was conducted on a total of 122 patients who underwent OLIF for SPL, lumbar spinal stenosis (LSS), foraminal stenosis (FS), and lumbar disc herniation (LDH) at our institution from July 2015 to June 2023. S-OLIF was started in November 2020 when an intraoperative surgical navigation system was installed. There were 61 patients in both the S-OLIF and D-OLIF groups. Patients who had previous fusion surgery at the index level (S-OLIF, n = 2; D-OLIF, n = 1) were excluded. Additionally, those who underwent multilevel OLIF (S-OLIF, n = 3; D-OLIF, n = 13) and one patient who underwent fluoroscopy-guided S-OLIF were excluded. Consequently, a total of 55 patients in the S-OLIF group and 47 patients in the D-OLIF group were included in the analysis (Supplementary Fig. S1). Demographic data, including age, sex, body mass index (BMI), diagnosis, clinical symptoms, surgery level, and comorbidities, were collected through retrospective medical record review. Follow-up visits were scheduled at 1, 3, 6, 12, and 24 months after surgery. Preoperative spine X-rays, computed tomography (CT), and magnetic resonance imaging (MRI) were performed for all patients. Immediate postoperative MRI was conducted after surgery, and spine X-rays were taken at each follow-up visit, with CT done at 6 or 12 months postoperatively. During outpatient visits, patients completed self-reports for the Oswestry disability index (ODI)^13^, EuroQol 5-dimension 5-Level (EQ-5D-5L) index^14^, and numeric rating scale (NRS)^15^ for both leg and low back pain. Surgical parameters, including total anesthesia time, total surgery time, time from anesthesia to skin incision, and estimated blood loss (EBL), were collected from anesthesia records. The institutional review board of Seoul National University Hospital waived the requirement for informed consent and approved the study protocol and chart review. All investigations were conducted in accordance with our institutional review board of Seoul National University Hospital guidelines and regulations. (Approval No. 2101-080-1187).

### Single-position oblique lumbar interbody fusion with navigation

Patients were positioned in the right lateral decubitus position with their hips and knees flexed at approximately 30 degrees. An intraoperative CT was conducted using the O-arm™ surgical imaging system (Medtronic, Minneapolis, MN, USA) after inserting the navigation reference frame at the left posterior superior iliac spine (PSIS). The images were then transferred to the StealthStation™ S8 navigation system (Medtronic, Minneapolis, MN, USA) for guidance during the surgery. Pedicle screws were inserted first, followed by an anterior approach^16^. The surgical approach involved making a skin incision and tapping after verifying the entry point for the pedicle screw using the navigation probe. Pedicle screws were inserted under the guidance of the navigation system. The navigation probe was used to confirm the index disc. For levels above L5, a 6 cm oblique skin incision was made anteriorly and posteriorly from the anterior margin of the index disc. The external oblique, internal oblique, and transverse abdominal muscles were dissected, and the endoabdominal fascia was gently detached from the iliac crest to access the retroperitoneal space. Major structures like the peritoneum, ureter, common iliac artery, and vein were protected and retracted anteriorly. The psoas muscle was detached from the vertebra and retracted posteriorly to expose the mid-point of the index disc. For the L5-S1 level, an oblique skin incision of 6 cm is made two finger-breadths anterior to the anterior superior iliac spine (ASIS), between the vertical line projected perpendicular to the floor from mid-point of the L5-S1 disc pace and the extension line of the S1 upper endplate. Avoiding injury to the abdominal wall nerves (especially the iliohypogastric and ilioinguinal nerves) and repairing the aponeurosis of the abdominal muscles are important to prevent abdominal wall hernia. If the inferior epigastric vessel is encountered during the approach, it could be ligated; however, the iliohypogastric and ilioinguinal nerves should not be ligated. A notable difference is the absence of the three-layered muscles (external oblique, internal oblique, and transverse abdominal muscle) in the lower abdominal wall. For the approach to L2-L5 from the flank, the three layers of muscle are split to approach the retroperitoneal space, and an approximation of the split muscles with sutures is sufficient for closure. However, for the L5-S1 approach, the aponeurosis of the three muscles is joined, and these layers are often not easy to discern and may need to be split as one or two layers. These layers should be very tightly closed with sutures to prevent abdominal hernia. Additionally, the L5-S1 disc space is approached between the left and right common iliac vessels to avoid injury to the iliac vein, its branches, and the lumbar plexus^17^. The differences in skin incision and approach for the two procedures are presented in Supplementary Fig. S2. After annulotomy, the disc was removed, and endplate preparation was done. A trial cage was inserted into the disc space under navigation guidance. The final case was filled with an appropriate fusion facilitating material and it was inserted, targeting the anterior one-third of the disc space. Alignment reduction was achieved using the previously placed pedicle screws, and the surgical wounds were closed.

### Dual-position oblique lumbar interbody fusion with fluoroscopy

The patients were placed in the same position as in S-OLIF, and the index disc was confirmed using C-arm fluoroscopy. In D-OLIF, the anterior approach was performed first, followed by the insertion of pedicle screws. Following a similar procedure as S-OLIF, the skin was incised, muscles and fascia were dissected, and retractors were placed to expose the mid-point of the index disc. Disc removal and endplate preparation were performed, followed by sequential insertion of the trial cage and final cage filled with fusion facilitating materials, all guided by C-arm fluoroscopy. Then, the abdominal wound was closed, and the patient was repositioned from the lateral to the prone position for pedicle screw insertion. Pedicle screws were inserted under the guidance of C-arm fluoroscopy. Alignment reduction was carried out, and the wound was closed.

### Clinical outcomes

Pain relief and functional improvement were evaluated by assessing changes in NRS-back, NRS-leg, ODI, EQ-5D-5L index, and walking more than 15 min without neurogenic intermittent claudication (NIC). Walking distance was measured by converting 15 min of walking into 1 km. Efficiency of operative time was analyzed by comparing total anesthesia time, total surgery time, and time from anesthesia to skin incision. Postoperative outcomes included a comparison of the types and rates of complications between the S-OLIF and D-OLIF groups. Additionally, the learning curve of S-OLIF was evaluated by comparing total surgery time, EBL, cumulative incidence of complications, and pedicle screw misplacement.

### Radiologic outcomes

The LL was measured by determining the Cobb angle between the upper endplate of L1 and the upper endplate of S1^18,19^. Similarly, the SL was measured by determining the Cobb angle between the upper endplate of the upper vertebral body at the index level and the lower endplate of the lower vertebral body at the index level^19^. Sagittal balance was evaluated by drawing the C7 plumb line and measuring the vertical distance between the posterior superior corner of S1 and the C7 plumb line^20^. Sagittal vertical axis (SVA) imbalance was defined as exceeding -40 mm to + 40 mm^21,22^. Changes in LL and SL was compared by measuring the angles on standing X-rays taken before and after the surgery. Additionally, the improvement of sagittal balance was compared. For cases of SPL, improvement in vertebral body slippage was evaluated by measuring changes in the vertebral body slippage ratio based on the upper endplate of the lower vertebral body before and after surgery^23^. The improvement in the foraminal area was evaluated using a spine MRI taken immediately after the surgery. During the follow-up period after surgery, screw accuracy was evaluated using spine CT and screw misplacement was assessed using the Gertzbein-Robbins grading scale^24^. The interbody fusion grade was evaluated according to the criteria proposed by Bridwell et al.’s study^25^, with fusion grade 1 and 2 being defined as successful fusion^26^. The disc height was measured by averaging the anterior border disc height and posterior border disc height^27,28^. Case malposition was defined as the situation where the center of the cage is outside the anterior one-third of the disc space^29^. Additionally, cage subsidence was defined as the presence of cortical bone destruction of 2 mm or more at any endplate, as observed on follow-up spine CT^30,31^. Instrument failure was evaluated based on the occurrence of screw loosening, screw fracture, and rod fracture observed on follow-up spine X-rays and spine CT^32^.

### Statistics

All statistical analyses were performed using SPSS version 26.0 (IBM Corp., Armonk, New York, USA), and a P value < 0.05 was considered statistically significant. Continuous variables were compared using an independent *t*-test, and the significance of categorical variables was tested using either the chi-square test or Fisher’s exact test. Scatter plots and cumulative distribution were analyzed using linear regression or non-linear regression, with the goodness of fit evaluated using the R-squared value. The regression model that best represented the data trend and had the highest R-squared value was selected to create the regression graph.

## Results

Out of a total of 102 patients, there were 55 patients in the S-OLIF group and 47 patients in the D-OLIF group. The mean age of patients in the S-OLIF group was 66.6 ± 9.7 years, while in the D-OLIF group, it was 67.8 ± 9.2 years. Among the patients, 38 (69.1%) and 28 (59.6%) were female, respectively. Due to S-OLIF being initiated at our institution in November 2020, the follow-up periods for the two groups were different (P < 0.001). In both groups, the most frequent diagnosis was SPL and among them, grade 1 was the most prevalent. The most common surgical level was L4-5. There were no significant differences in BMI, current smoker ratio, osteoporosis, and diabetes mellitus between the two groups. The baseline characteristics of the patients are presented in Table 1.

**Table 1:** Demographics of patients.

	S-OLIF (n = 55)	D-OLIF (n = 47)	P value
Age (year), mean ± SD (range)	66.6 ± 10.1 (21–84)	67.8 ± 9.2 (49–88)	0.525
Female, n (%)	38 (69.1)	28 (59.6)	0.316
Follow-up period (months), mean ± SD (range)	9.6 ± 6.0 (10.7–24.5)	17.8 ± 8.0 (3.0–28.7)	< 0.001
Diagnosis, n (%)			
Spondylolisthesis	48 (87.3)	27 (57.5)	0.001
Lumbar disc herniation	1 (1.8)	3 (6.4)	0.332
Lumbar spinal stenosis	5 (9.1)	14 (29.8)	0.007
Foraminal stenosis, n (%)	3 (5.5)	1 (2.1)	0.332
Spondylolisthesis grade 1, n (%)	31 (64.6, n = 48)	24 (88.9, n = 27)	0.022
Spondylolisthesis grade 2, n (%)	16 (33.3, n = 48)	3 (11.1, n = 27)	0.034
Spondylolisthesis grade 3, n (%)	1 (1.8, n = 48)	0 (0, n = 27)	1.000
Surgery level, n (%)			
L2-3	0 (0)	1 (2.1)	0.461
L3-4	4 (7.3)	6 (12.8)	0.507
L4-5	39 (70.9)	23 (48.9)	0.023
L5-S1	12 (21.8)	17 (36.2)	0.109
History of previous posterior decompression surgery	2 (3.6)	2 (4.3)	1.000
Body mass index (kg/m^2^), mean ± SD (range)	24.5 ± 3.7 (15.6–37.4)	24.9 ± 2.9 (19.2–32.9)	0.592
Current smoker, n (%)	3 (5.5)	5 (10.6)	0.465
Osteoporosis, n (%)	9 (28.2, n = 32)	8 (24.2, n = 33)	0.722
Diabetes mellitus, n (%)	10 (18.2)	14 (29.8)	0.168

*S-OLIF* single-position oblique lumbar interbody fusion with navigation, *D-OLIF* dual-position oblique lumbar interbody fusion with fluoroscopy, *SD* standard deviation.

### Comparison of clinical outcomes between two groups

**Figure 1:**
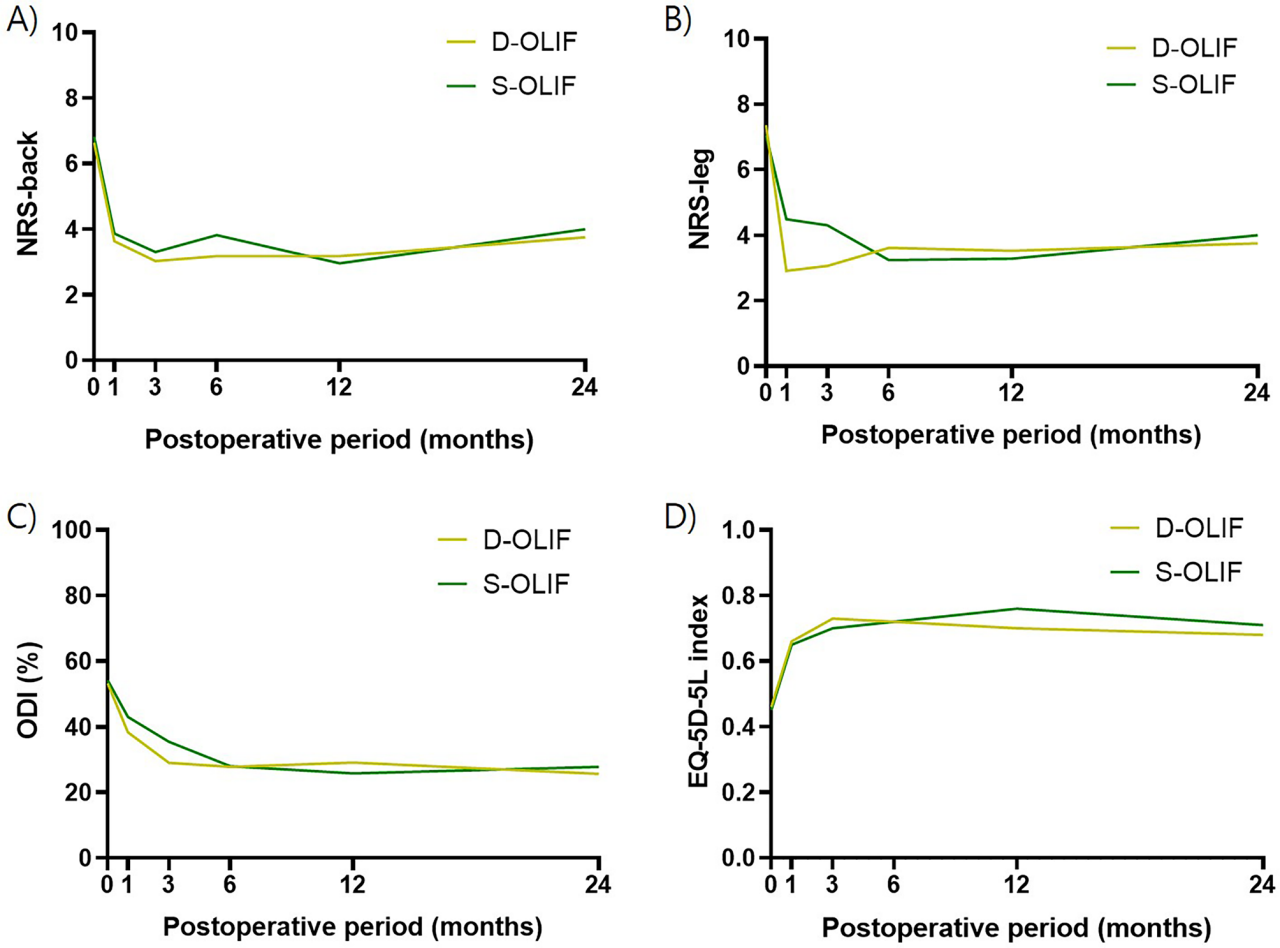
Serial follow-up of patents reported clinical outcomes. (**A**) The graph represents the numeric rating scale (NRS) for back pain in single-position oblique lumbar interbody fusion with navigation (S-OLIF) and dual-position oblique lumbar interbody fusion with fluoroscopy (D-OLIF) patients at preoperative and postoperative follow-ups (1 month, 3 months, 6 months, 12 months, and 24 months). (**B**) The graph represents the NRS for leg pain in S-OLIF and D-OLIF patients at preoperative and postoperative follow-ups (1 month, 3 months, 6 months, 12 months, and 24 months). (**C**) The graph represents the Oswestry disability index (ODI) in S-OLIF and D-OLIF patients at preoperative and postoperative follow-ups (1 month, 3 months, 6 months, 12 months, and 24 months). (**D**) The graph represents the EuroQol 5-dimension 5-Level (EQ-5D-5L) index in S-OLIF and D-OLIF patients at preoperative and postoperative follow-ups (1 month, 3 months, 6 months, 12 months, and 24 months).

In both groups, NRS-back, NRS-leg, ODI, and walking more than 15 min without NIC improved from preoperative to the last follow-up, but there were no significant differences between the two groups (P = 0.749, 0.148, 0.677, and 0.150, respectively). Conversely, the EQ-5D-5L index improved in both groups from preoperative to the last follow-up, with a higher score in the S-OLIF group at 0.75 ± 0.12 compared to 0.67 ± 0.18 in the D-OLIF group (P = 0.029). The serial changes in clinical parameters during preoperative and follow-up periods are presented in Fig. 1. There were 19 complications (34.6%) and 11 complications (23.4%) in each group, but there was no significant difference between the two groups (P = 0.115). Only the incidence of genitofemoral nerve irritation signs was higher in the S-OLIF group than in the D-OLIF group (P = 0.019). There were no cases of large vessel injury, ureter injury, hernia, or secondary surgery during the follow-up period in either group. The specific complications, including symptoms, treatments, and outcomes, that occurred in the S-OLIF and D-OLIF groups are presented in Supplementary Table S1. The length of hospital stay was little longer in the S-OLIF group, with 4.9 ± 1.6 days compared to 4.0 ± 1.0 days in the D-OLIF group (P = 0.001). The clinical outcomes of the patients are presented in Table 2. Additionally, the clinical outcomes of the three patients who underwent multilevel S-OLIF are presented in Supplementary Table S2.

**Table 2:** Comparison of clinical outcomes between single-position oblique lumbar interbody fusion with navigation and dual-position oblique lumbar interbody fusion with fluoroscopy.

	S-OLIF (n = 55)	D–OLIF (n = 47)	P value
NRS–back, mean ± SD (range)			
Preoperative	6.8 ± 2.3 (0–10)	6.6 ± 2.7 (0–10)	0.713
Last follow-up	3.3 ± 2.1 (0–8) (n = 54)	3.1 ± 2.4 (0–8) (n = 39)	0.749
NRS–leg, mean ± SD (range)			
Preoperative	7.0 ± 2.1 (0–10)	7.4 ± 2.6 (0–10)	0.492
Last follow-up	3.2 ± 2.3 (0–8) (n = 54)	4.0 ± 2.7 (0–8) (n = 39)	0.148
ODI (%), mean ± SD (range)			
Preoperative	54.2 ± 17.3 (17.8–86.7)	53.3 ± 19.4 (20–91.1)	0.797
Last follow-up	28.2 ± 14.7 (2.2–64.4) (n = 51)	29.6 ± 16.6 (6.7–68.9) (n = 39)	0.677
EQ-5D-5L index, mean ± SD (range)			
Preoperative	0.45 ± 0.19 (0–0.83)	0.46 ± 0.24 (0.05–0.85) (n = 45)	0.891
Last follow-up	0.75 ± 0.12 (0.38–0.90) (n = 54)	0.67 ± 0.18(0.03–0.86) (n = 37)	0.029
Walking more than 15 min without NIC, n (%)			
Preoperative	5 (10, n = 50)	9 (22.5, n = 40)	0.104
Last follow-up	37 (92.5, n = 40)	10 (76.9, n = 13)	0.150
Complications, n (%)	19 (34.6)	11 (23.4)	0.218
Ipsilateral transient psoas weakness	2 (3.6)	1 (2.1)	1.000
Peritoneal injury	1 (1.8)	1 (2.1)	1.000
Paralytic ileus	2 (3.6)	2 (4.3)	1.000
Ipsilateral sympathetic chain symptoms	5 (9.1)	6 (12.8)	0.551
Irritation of ipsilateral genitofemoral nerve	9 (16.4)	1 (2.1)	0.019
Large vessel injury	0 (0)	0 (0)	
Ureter injury	0 (0)	0 (0)	
Hernia	0 (0)	0 (0)	
Secondary surgery, n (%)	0 (0)	0 (0)	
Length of hospital stay (day), mean ± SD (range)	4.9 ± 1.6 (3–12)	4.0 ± 1.0 (3–6)	0.001

*S-OLIF* single-position oblique lumbar interbody fusion with navigation, *D-OLIF* dual-position oblique lumbar interbody fusion with fluoroscopy, *NRS* numerical rating scale, *SD* standard deviation, *ODI* Oswestry disability index, *EQ-5D-5L* EuroQol 5-dimension 5-level, *NIC* neurogenic intermittent claudication.

### Comparison of radiological outcomes between two groups

**Figure 2:**
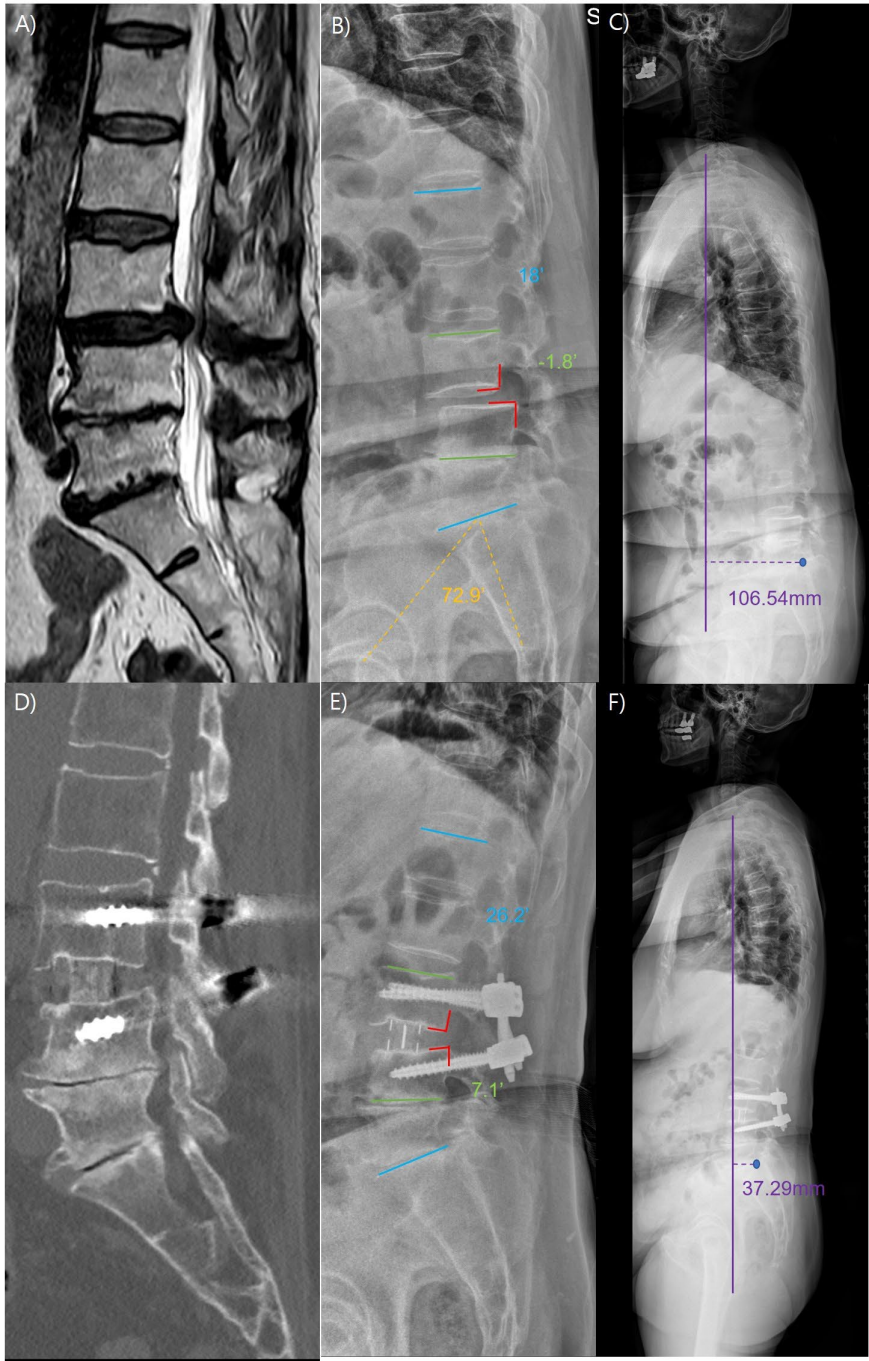
A representative case of improved radiological parameters in dual-position oblique lumbar interbody fusion with fluoroscopy. (**A**) The patient is a 75-year-old female with spondylolisthesis (SPL) grade 1 at the L3-4 level. (**B** and **C**) Her pelvic incidence (PI) is measured at 72.9 degrees, while the lumbar lordosis (LL) is 18 degrees, resulting in a PI-LL mismatch of 54.9 degrees. The segmental lordosis (SL) at L3-4 is -1.8 degrees, and positive sagittal imbalance is evident. (**D**) The patient underwent a dual-position oblique lumbar interbody fusion with fluoroscopy (D-OLIF). A computed tomography (CT) performed one year after surgery confirmed cage subsidence, but showed successful fusion and no instrument failure. (**E** and **F**) The postoperative one-year follow-up X-ray shows that vertebral body slippage has been reduced. The LL measures 26.2 degrees. The SL has increased to 7.1 degrees, and sagittal imbalance has improved.

**Figure 3:**
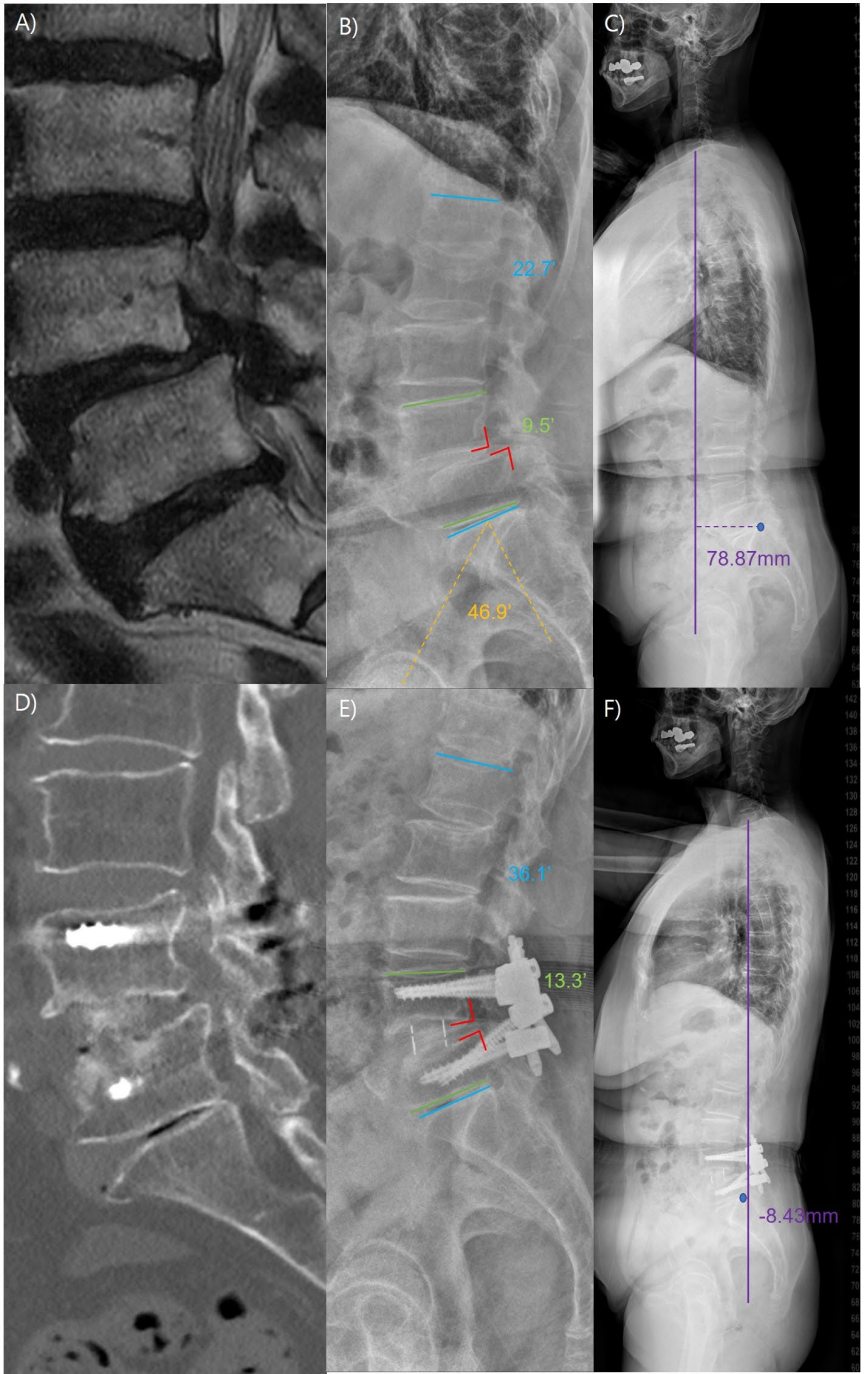
A representative case of improved radiological parameters in single-position oblique lumbar interbody fusion with navigation. (**A**) The patient is a 77-year-old female with spondylolisthesis (SPL) grade 1 at the L4-5 level. (**B** and **C**) Her pelvic incidence (PI) is measured at 46.9 degrees, while the lumbar lordosis (LL) is 22.7 degrees, resulting in a PI-LL mismatch of 24.2 degrees. The segmental lordosis (SL) at L4-5 is 9.5 degrees, and positive sagittal imbalance is evident. (**D**) The patient underwent single-position oblique lumbar interbody fusion with navigation (S-OLIF). Computed tomography (CT) performed one year one year after surgery confirmed successful fusion, no instrument failure, and no cage subsidence. (**E** and **F**) The postoperative one-year follow-up X-ray shows that vertebral body slippage has been reduced. The LL measures 36.1 degrees. The SL has increased to 13.1 degrees, and sagittal imbalance has improved.

Postoperatively, the bilateral foraminal area expanded in both groups, and disc height increased at the last follow-up for both groups, but there were no significant differences between the two groups (P = 0.881, 0.980, and 0.131, respectively). SL increased to 16.6 ± 8.7 degrees in the S-OLIF group (preoperative; 11.7 ± 8.6) and 17.6 ± 8.5 degrees in the D-OLIF group (preoperative; 13.4 ± 8.9). The LL increased to 47.6 ± 13.4 degree in the S-OLIF group (preoperative; 42.1 ± 15.3) and 47.9 ± 13.8 degree in the D-OLIF group (preoperative; 42.8 ± 14.1). However, there was no significant difference between the two groups (P = 0.536, and 0.914, respectively). C7 SVA imbalance improved in both groups and especially in SPL patients, vertebral body slippage improved in both groups (P = 0.617 and 0.784). Figure 2 presented a representative case of D-OLIF showing the improvement in radiologic parameters. Figure 3 presented a representative case of S-OLIF showing the improvement in radiologic parameters. There were no cases of cage malposition in either group. Overall, pedicle screw misplacement was significantly higher in the D-OLIF group (8 out of 92, 8.70%) compared to the S-OLIF group (2 out of 144, 1.39%) (P = 0.045). Notably, in the S-OLIF group, all misplacements were cortical breaches less than 2 mm, whereas in the D-OLIF group, the majority (7 out of 8, 87.5%) had cortical breaches greater than 2 mm. The radiological outcomes of the patients are presented in Table 3. Additionally, the radiological outcomes of the three patients who underwent multilevel S-OLIF are presented in Supplementary Table S3.

**Table 3:** Comparison of radiological outcomes between single-position oblique lumbar interbody fusion with navigation and dual-position oblique lumbar interbody fusion with fluoroscopy.

	S-OLIF (n = 55)	D-OLIF (n = 47)	P value
Foraminal area, right (mm^2^), mean ± SD (range)			
Preoperative	50.6 ± 14.3 (24.6–86.6) (n = 54)	52.2 ± 13.3 (25.9–91.6)	0.553
Postoperative	70.8 ± 22.6 (35.6–130.4) (n = 52)	71.6 ± 21.5 (42.4–132.4) (n = 28)	0.881
Foraminal area, left (mm^2^), mean ± SD (range)			
Preoperative	53.9 ± 15.0 (27.6–90.4) (n = 54)	54.5 ± 14.6 (29.3–90.6)	0.828
Postoperative	77.7 ± 21.0 (42.0–134.1) (n = 52)	77.8 ± 19.0 (42.8–132.4) (n = 28)	0.980
Disc height (mm), mean ± SD (range)			
Preoperative	6.5 ± 3.1 (0.2–13.7)	7.9 ± 3.6 (0.7–19.7)	0.043
Last follow-up	13.3 ± 3.3 (7.2–27.6)	12.4 ± 2.1 (7.1–17.2)	0.131
Segmental lordosis (degree), mean ± SD (range)			
Preoperative	11.7 ± 8.6 (-8.1–29.1)	13.4 ± 8.9 (– 6.8–34.7)	0.333
Last follow-up	16.6 ± 8.7 (-7.2–36.5)	17.6 ± 8.4 (– 1.8–38.7)	0.536
Lumbar lordosis (degree), mean ± SD (range)			
Preoperative	42.1 ± 15.3 (9.4–74.8)	42.8 ± 14.1 (9.0–64.9)	0.806
Last follow-up	47.6 ± 13.4 (21.7–84.6)	47.9 ± 13.8 (21.5–77.2)	0.914
C7 sagittal vertical axis imbalance, n (%)			
Preoperative	24 (43.6)	15 (31.9)	0.225
Last follow-up	8 (25.8)	8 (25.8, n = 31)	0.617
Vertebral body slippage (%), mean ± SD (range)			
Preoperative	22.6 ± 9.5 (4.3–55.8) (n = 48)	16.5 ± 9.5 (1.3–37.0) (n = 27)	0.010
Last follow-up	8.0 ± 3.7 (2.0–19.4) (n = 48)	7.6 ± 5.9 (0.3–22.9) (n = 27)	0.784
Successful interbody fusion, n (%)	32 (88.9, n = 36)	20 (87.0, n = 23)	1.000
Case malposition, n (%)	0 (0)	0 (0, n = 41)	
Cage subsidence, n (%)	10 (27.8, n = 36)	8 (34.8, n = 23)	0.569
Pedicle screw misplacement, n (%)	2 (1.4, n = 144)	8 (8.7, n = 92)	0.045
< 2 mm cortical breach	2 (100, n = 2)	1 (12.5, n = 8)	0.067
< 4 mm cortical breach	0 (0, n = 2)	5 (62.5, n = 8)	0.444
< 6 mm cortical breach	0 (0, n = 2)	2 (25, n = 8)	1.000
> 6 mm cortical breach	0 (0, n = 2)	0 (0, n = 8)	
Instrument failure, n (%)	4 (7.3)	2 (4.3)	0.684

*S-OLIF* single-position oblique lumbar interbody fusion with navigation, *D-OLIF* dual-position oblique lumbar interbody fusion with fluoroscopy, *SD* standard deviation.

### Operative time efficiency of single-position oblique lumbar interbody fusion with navigation

After anesthesia and before the skin incision, a navigation CT was taken. Consequently, the time between anesthesia to skin incision was longer in the S-OLIF group, with 61.7 ± 14.6 minutes compared to 54.1 ± 14.5 minutes in the D-OLIF group (P = 0.010). However, the total anesthesia time and total surgery time were shorter in the S-OLIF group (P = 0.006 and 0.002, respectively). The surgical parameters of the patients are presented in Table 4.

**Table 4:** Surgical parameters of single-position oblique lumbar interbody fusion with navigation and dual-position oblique lumbar interbody fusion with fluoroscopy.

	S-OLIF (n = 55)	D-OLIF (n = 47)	P value
Total anesthesia time (minute), mean ± SD (range)	253.7 ± 39.8 (180–385)	285.7 ± 68.5 (180–450)	0.006
Total surgery time (minute), mean ± SD (range)	183.6 ± 34.3 (125–275)	216.0 ± 9.2 (125–350)	0.002
Time between anesthesia to skin incision (minute), mean ± SD (range)	61.7 ± 14.6 (25–100)	54.1 ± 14.5 (35–105)	0.010
Estimated blood loss (ml), mean ± SD (range)	167.1 ± 99.6 (20–450)	175.0 ± 115.2 (50–500) (n = 44)	0.715

*S-OLIF* single-position oblique lumbar interbody fusion with navigation, *D-OLIF* dual-position oblique lumbar interbody fusion with fluoroscopy, *SD* standard deviation.

### Learning curve of single-position oblique lumbar interbody fusion with navigation

**Figure 4:**
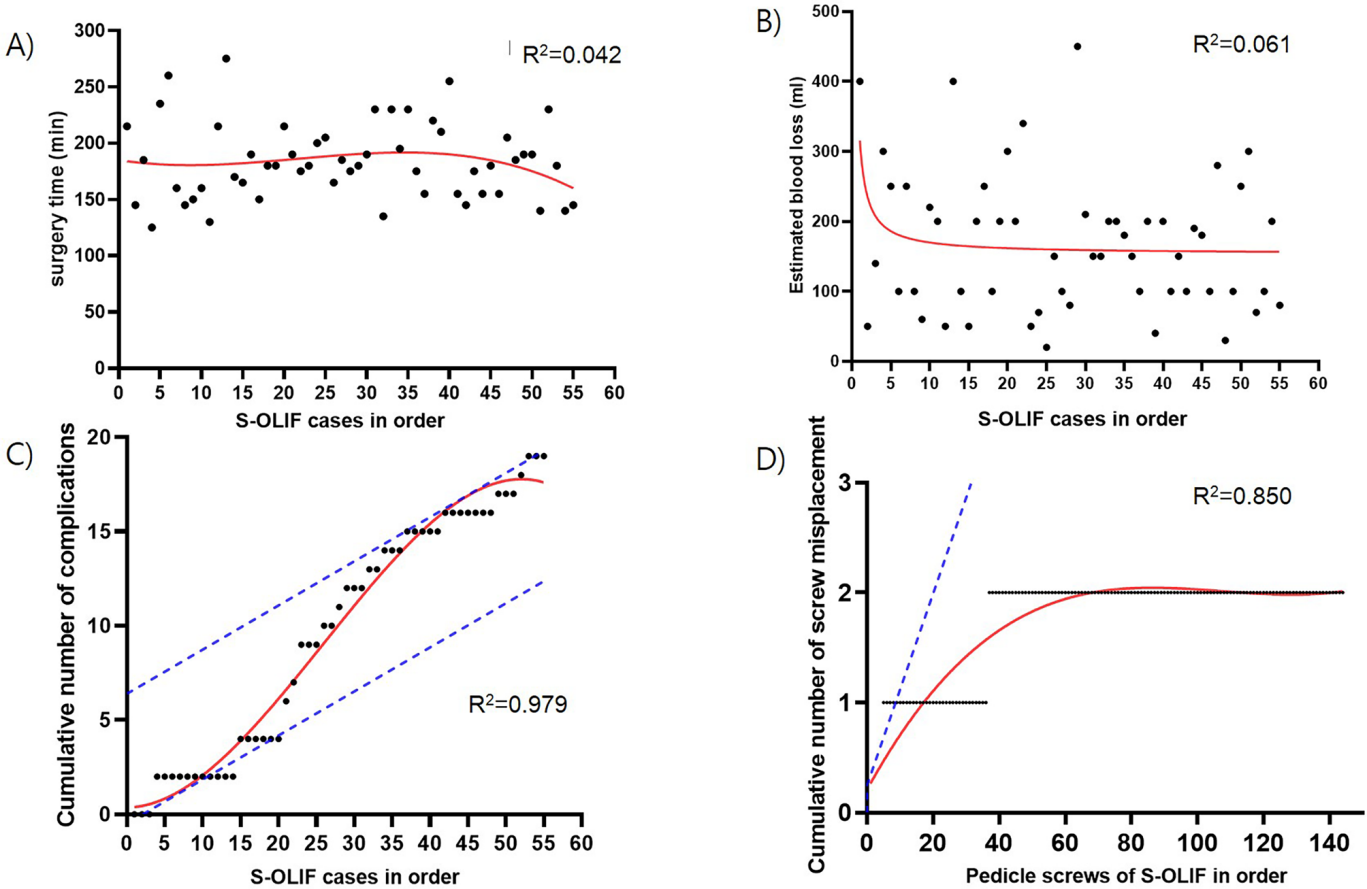
Learning curve parameters of single-position oblique lumbar interbody fusion with navigation. (**A**) The scatter plot shows the single-position oblique lumbar interbody fusion with navigation (S-OLIF) cases over time and their corresponding surgery times. The non-linear regression with third order polynomial model is shown in red (R^2^ = 0.042). As the number of cases accumulated, the overall trend of surgery time showed a slight decrease, but there was no statistical significance (P = 0.527). (**B**) The scatter plot shows the S-OLIF cases over time and their corresponding estimated blood loss (EBL). The non-linear regression with the inverse model is shown in red (R^2^ = 0.061). As the number of cases accumulated, the overall trend of EBL showed a decrease, but there was no statistical significance (P = 0.069). (**C**) The scatter plot shows the S-OLIF cases over time and their corresponding cumulative complication count. The non-linear regression with third order polynomial model is shown in red (R^2^ = 0.979, P < .001). The cumulative complication rate for dual-position oblique lumbar interbody fusion with fluoroscopy (D-OLIF) was depicted as a blue dashed line, and it was parallelly shifted to intersect with the cumulative complication curve for S-OLIF. After the 10th case of S-OLIF, the cumulative complication rate increased compared to the cumulative complication rate of D-OLIF. However, from the 41st case of S-OLIF onwards, the cumulative complication rate showed a decreasing trend. (**D**) The scatter plot shows the pedicle screws of S-OLIF cases over time and their corresponding cumulative screw misplacement count. The non-linear regression with third order polynomial model is shown in red (R^2^ = 0.850, P < 0.001). The cumulative screw misplacement rate for D-OLIF was depicted as a blue dashed line, and it was parallelly shifted to intersect with the cumulative screw misplacement curve for S-OLIF. The screw misplacement rate in S-OLIF showed a lower trend compared to the cumulative screw misplacement rate of D-OLIF from the early cases of S-OLIF onwards.

The scatter plot of the S-OLIF group, arranged in chronological order from the first case, displayed a decreasing trend in surgery time and EBL. However, these trends were not statistically significant (P = 0.527 and 0.069, respectively). Initially, S-OLIF demonstrated a lower cumulative complication rate compared to D-OLIF. However, in the mid-cases, S-OLIF showed a higher trend in cumulative complication occurrence than D-OLIF. From the 41st case onwards, it became similar to D-OLIF's cumulative complication rate and showed a decreasing trend thereafter. (P < 0.001) The screw misplacement rate in S-OLIF was lower than the cumulative screw misplacement rate of D-OLIF from the early cases. (P < 0.001) The scatter plots and non-linear regression results for surgery time, EBL, cumulative complications, and cumulative screw misplacement were presented in Fig. 4.

## Discussion

In this study, we compared clinical and radiological outcomes between S-OLIF and D-OLIF. At the last follow-up, the S-OLIF group showed significantly better functional improvement based on the EQ-5D-5L index. However, there were no significant differences in other outcome measurements between the two groups. The S-OLIF group had a slightly longer hospitalization period due to two specific cases with paralytic ileus and genitofemoral nerve irritation, respectively. Overall complication occurrence did not differ significantly between the groups, but S-OLIF showed a higher incidence of genitofemoral nerve irritation signs. The use of navigation in S-OLIF might have led to increased psoas muscle retraction compared to D-OLIF because it provides real-time feedback on the center point of the disc. There were no significant differences in measurements of radiological outcomes. Specifically, SL and LL increased in both groups after surgery, but there were no significant differences between the two groups. However, the accuracy of pedicle screw placement was higher in the S-OLIF group.

### General concept and strength of oblique lumbar interbody fusion

OLIF is one of the approaches used in lateral lumbar interbody fusion (LLIF), involving access to the retroperitoneal space through the gap between the anterior vessels and the psoas muscle^33^. The interbody device utilized in OLIF allows for effective distraction to achieve foraminal decompression, ensuring the acquisition of a lordotic angle and promoting rapid fusion^34^. When compared to another LLIF known as direct lumbar interbody fusion (DLIF), OLIF has demonstrated a reduction in the risks of psoas muscle injury and lumbar plexus injury, along with improved sagittal balance correction^35,36^. Moreover, OLIF has shown a lower incidence of sympathetic chain injury and major vessel injury compared to anterior lumbar interbody fusion (ALIF)^36,37^. In comparison to transforaminal lumbar interbody fusion (TLIF), OLIF has shown no difference in patient-reported outcomes. However, OLIF exhibits superior disc height restoration and faster fusion. Additionally, OLIF requires a shorter surgical duration and results in lower EBL when compared to minimally-invasive TLIF^38^.

### Advantages of using intraoperative navigation in fusion surgery

The use of navigation in S-OLIF offers several advantages, including precise and accurate insertion of cages and pedicle screws^39,40^. Real-time feedback provided by the navigation system allows surgeons to follow optimal trajectories and depths, reducing the risk of instrument malposition or misplacement and improving overall surgical outcomes^41^. Notably, a study reporting on cage insertion with navigation guidance achieved an impressive success rate of 94.86% in achieving appropriate cage placement^40^. Additionally, when using navigation for pedicle screw insertion, the reported accuracy rate was 90.76%, surpassing the accuracy of pedicle screw placement guided by fluoroscopy at 85.48%^42^. Furthermore, when pedicle screws were inserted using navigation guidance in the lateral position, the screw malposition rate was as low as 1.8% to 3.5%^2,7^. In contrast, D-OLIF requires patient repositioning from lateral to prone position for pedicle screw insertion, leading to longer surgery times and potentially increased pressure on anterior elements, raising the risk of complications^43–45^. In this study, a single spine neurosurgeon performed both the anterior approach for cage insertion and the pedicle screw insertion sequentially. However, simultaneous procedures by two surgeons, with one focusing on the anterior approach and the other on the posterior approach, can further shorten the surgery time^2^. Additionally, navigation can be particularly beneficial in cases of severe obesity or significant degenerative changes, where identifying anatomical landmarks using fluoroscopy is challenging^46^. To accurately guide the insertion of the cage and pedicle screw using fluoroscopy, orthogonal views are required. As a result, frequent fluoroscopy image acquisition is essential during the process of cage and pedicle screw insertion^41,47^. However, this frequent fluoroscopy imaging can increase radiation exposure for both the surgeon and the patient^48^. On the other hand, the utilization of navigation can reduce the reliance on fluoroscopy during cage and pedicle screw insertion, thereby decreasing radiation exposure for the surgeon and the patient^48^. In cases where pedicle screw insertion is performed in the lateral position, concerns about insufficient SL and LL compared to the prone position have been raised^12^. However, several studies have reported that repositioning from the lateral position to the prone position during LLIF does not result in a significant increase in LL^41–49^. Ouchida et al.^2^ conducted a comparison of LL generated in single-position LLIF and repositioning interbody fusion. In single-position LLIF, the gain in LL was measured to be 4.2 degrees. After repositioning the patient to the prone position for pedicle screw insertion, the obtained LL was 4.4 degrees. The study found no significant difference in lumbar lordosis between the two groups.

### Learning curve of single-position oblique lumbar interbody fusion with navigation

Warren et al.^43^ reported in a retrospective analysis of single-position LLIF that as the number of surgical cases increased, the surgical time decreased. Additionally, they found no significant differences in complications or clinical outcomes when comparing it to dual-position LLIF. Ultimately, the study suggested that surgeons with prior experience in dual-position LLIF may encounter a steeper learning curve when performing single-position LLIF. Blizzard et al.^51^ reported a retrospective analysis of minimally-invasive single-position LLIF or OLIF and similarly reported that surgeons with prior experience in the traditional prone position technique for pedicle screw insertion may not face a significant learning curve when inserting pedicle screws in the lateral position. In this study, S-OLIF procedures were performed by surgeons experienced in D-OLIF. The S-OLIF group showed a mean surgery time of 32.4 min shorter than D-OLIF. However, when analyzing the trend of surgery time in S-OLIF cases chronologically, there was a slight decreasing trend, but it was not statistically significant. Similarly, the trend of EBL over time in S-OLIF cases showed a decrease, but it was not statistically significant either. The cumulative complication rate of S-OLIF initially had a lower occurrence rate compared to D-OLIF in the early cases. However, it increased in the mid-cases and then decreased in the later cases, but it did not precisely follow the concept of a learning curve for complication occurrence. Regarding pedicle screw accuracy, S-OLIF had a lower occurrence rate of screw misplacement compared to D-OLIF from the early cases. Based on these findings, it can be concluded that surgeons experienced in D-OLIF may not require a significant learning curve or only a limited number of cases when performing S-OLIF, which aligns with conclusions drawn in previous studies on learning curves.

### Limitations

This retrospective analysis of prospective collected data was conducted at a single institution, and the sample size of the study subjects was limited. Therefore, future large-scale prospective studies with an increased number of subjects will be necessary. Additionally, due to the relatively short postoperative follow-up period, complications such as adjacent segment disease potentially arising from fusion could not be evaluated. Lastly, since S-OLIF was performed by surgeons with prior experience in D-OLIF, the learning curve for S-OLIF in surgeons without prior D-OLIF experience could not be evaluated.

## Conclusion

S-OLIF showed better functional outcomes compared to D-OLIF while achieving similar improvements in sagittal balance and complication occurrence. S-OLIF also demonstrated shorter surgery time and allowed for more accurate pedicle screw insertion compared to D-OLIF. Lastly, the learning curve for S-OLIF is almost non-existent or requires only a small number of cases for surgeons experienced in D-OLIF.

### Supplementary Information

Supplementary Figures.

Supplementary Tables.

## Data Availability

The datasets generated during and/or analyzed during the current study are available from the corresponding author upon reasonable request.

## References

[1] Kotani, Y., Koike, Y., Ikeura, A., Tokunaga, H. & Saito, T. Clinical and radiologic comparison of anterior-posterior single-position lateral surgery versus MIS-TLIF for degenerative lumbar spondylolisthesis. *J. Orthop. Sci.* **26**, 992–998. 10.1016/j.jos.2020.10.013 (2021). 33339720 10.1016/j.jos.2020.10.013

[2] Ouchida, J. *et al.* Simultaneous single-position lateral interbody fusion and percutaneous pedicle screw fixation using O-arm-based navigation reduces the occupancy time of the operating room. *Eur. Spine J.* **29**, 1277–1286. 10.1007/s00586-020-06388-6 (2020). 32239355 10.1007/s00586-020-06388-6

[3] Bassani, R. *et al.* Functional and radiological outcome of anterior retroperitoneal versus posterior transforaminal interbody fusion in the management of single-level lumbar degenerative disease. *Neurosurg. Focus FOC* **49**, E2. 10.3171/2020.6.Focus20374 (2020). 10.3171/2020.6.Focus20374 32871567

[4] Nomura, H., Yamashita, A., Watanabe, T. & Shirasawa, K. Quantitative analysis of indirect decompression in extreme lateral interbody fusion and posterior spinal fusion with a percutaneous pedicle screw system for lumbar spinal stenosis. *J. Spine Surg.* **5**, 266–272 (2019). 31380481 10.21037/jss.2019.06.03 PMC6626744

[5] Feeley, A., Feeley, I., Clesham, K. & Butler, J. Is there a variance in complication types associated with ALIF approaches? A systematic review. *Acta Neurochir.* **163**, 2991–3004. 10.1007/s00701-021-05000-0 (2021). 34546435 10.1007/s00701-021-05000-0 PMC8520518

[6] Woods, K. R. M., Billys, J. B. & Hynes, R. A. Technical description of oblique lateral interbody fusion at L1–L5 (OLIF25) and at L5–S1 (OLIF51) and evaluation of complication and fusion rates. *Spine J.* **17**, 545–553. 10.1016/j.spinee.2016.10.026 (2017). 27884744 10.1016/j.spinee.2016.10.026

[7] Kim, H. C. *et al.* Single-position oblique lumbar interbody fusion and percutaneous pedicle screw fixation under o-arm navigation: A retrospective comparative study. *J. Clin. Med.* **12**, 312 (2023). 10.3390/jcm12010312 PMC9821558 36615112

[8] Tan, Y. *et al.* Comparison of simultaneous single-position oblique lumbar interbody fusion and percutaneous pedicle screw fixation with posterior lumbar interbody fusion using o-arm navigated technique for lumbar degenerative diseases. *J. Clin. Med.*10.3390/jcm10214938 (2021). 34768459 10.3390/jcm10214938 PMC8584546

[9] Cheng, P., Zhang, X.-B., Zhao, Q.-M. & Zhang, H.-H. Efficacy of single-position oblique lateral interbody fusion combined with percutaneous pedicle screw fixation in treating degenerative lumbar spondylolisthesis: A cohort study. *Front. Neurol.*10.3389/fneur.2022.856022 (2022). 35785341 10.3389/fneur.2022.856022 PMC9240256

[10] Tanaka, M. *et al.* Comparison of C-arm-free oblique lumbar interbody fusion L5–S1 (OLIF51) with transforaminal lumbar interbody fusion L5–S1 (TLIF51) for adult spinal deformity. *Medicina* **59**, 838 (2023). 37241070 10.3390/medicina59050838 PMC10221075

[11] Zhang, X., Guo, Y. & Li, Y. Comparison of the clinical efficacy of two fixation methods combined with OLIF in the treatment of lumbar spondylolisthesis in adult patients. *J. Orthop. Surg. Res.* **17**, 115. 10.1186/s13018-022-02991-z (2022). 35189897 10.1186/s13018-022-02991-z PMC8862256

[12] Yson, S. C., Sembrano, J. N., Santos, E. R. G., Luna, J. T. P. & Polly, D. W. J. Does prone repositioning before posterior fixation produce greater lordosis in lateral lumbar interbody fusion (LLIF)?. *Clin. Spine Surg.* **27**, 364–369. 10.1097/BSD.0b013e318268007b (2014). 10.1097/BSD.0b013e318268007b 22801455

[13] Fairbank, J. C., Couper, J., Davies, J. B. & O’Brien, J. P. The Oswestry low back pain disability questionnaire. *Physiotherapy* **66**, 271–273 (1980). 6450426

[14] Kim, S. H. *et al.* The EQ-5D-5L valuation study in Korea. *Qual. Life Res.* **25**, 1845–1852. 10.1007/s11136-015-1205-2 (2016). 26961008 10.1007/s11136-015-1205-2

[15] Huskisson, E. C. Measurement of pain. *Lancet* **2**, 1127–1131. 10.1016/s0140-6736(74)90884-8 (1974). 4139420 10.1016/s0140-6736(74)90884-8

[16] Han, J. *et al.* Surgical treatment of spondylolisthesis by oblique lumbar interbody fusion and transpedicular screw fixation: Comparison between conventional double position versus navigation-assisted single lateral position. *PLoS One* **18**, e0291114. 10.1371/journal.pone.0291114 (2023). 37708151 10.1371/journal.pone.0291114 PMC10501584

[17] Orita, S. *et al.* Technical and conceptual review on the L5–S1 oblique lateral interbody fusion surgery (OLIF51). *Spine Surg. Relat. Res.* **5**, 1–9. 10.22603/ssrr.2020-0086 (2021). 33575488 10.22603/ssrr.2020-0086 PMC7870318

[18] Okpala, F. O. Comparison of four radiographic angular measures of lumbar lordosis. *J. Neurosci. Rural Pract.* **9**, 298–304. 10.4103/jnrp.jnrp_508_17 (2018). 30069082 10.4103/jnrp.jnrp_508_17 PMC6050761

[19] Kim, S. B. *et al.* Radiographic results of single level transforaminal lumbar interbody fusion in degenerative lumbar spine disease: Focusing on changes of segmental lordosis in fusion segment. *Clin. Orthop. Surg.* **1**, 207–213. 10.4055/cios.2009.1.4.207 (2009). 19956478 10.4055/cios.2009.1.4.207 PMC2784961

[20] Le Huec, J. C., Thompson, W., Mohsinaly, Y., Barrey, C. & Faundez, A. Sagittal balance of the spine. *Eur. Spine J.* **28**, 1889–1905. 10.1007/s00586-019-06083-1 (2019). 31332569 10.1007/s00586-019-06083-1

[21] Jia, J., Zhao, Y. & Liu, X. Impact of sagittal imbalance correction on clinical outcomes in patients undergoing MIS-TLIF for LSS. *Clin. Neurol. Neurosurg.* **181**, 119–126. 10.1016/j.clineuro.2019.04.017 (2019). 31039493 10.1016/j.clineuro.2019.04.017

[22] Teraguchi, M. *et al.* Sagittal imbalance of the spine-pelvis-lower extremity axis associated with back-related disability. *J. Orthop. Sci.* **26**, 986–991. 10.1016/j.jos.2020.10.014 (2021). 33293187 10.1016/j.jos.2020.10.014

[23] Koslosky, E. & Gendelberg, D. Classification in brief: The Meyerding classification system of spondylolisthesis. *Clin. Orthop. Relat. Res.* **478**, 1125–1130. 10.1097/corr.0000000000001153 (2020). 32282463 10.1097/corr.0000000000001153 PMC7170696

[24] Gertzbein, S. D. & Robbins, S. E. Accuracy of pedicular screw placement in vivo. *Spine (Phila Pa 1976)* **15**, 11–14. 10.1097/00007632-199001000-00004 (1990). 2326693 10.1097/00007632-199001000-00004

[25] Bridwell, K. H., Lenke, L. G., McEnery, K. W., Baldus, C. & Blanke, K. Anterior fresh frozen structural allografts in the thoracic and lumbar spine. Do they work if combined with posterior fusion and instrumentation in adult patients with kyphosis or anterior column defects?. *Spine (Phila Pa 1976)* **20**, 1410–1418 (1995). 7676341 10.1097/00007632-199506020-00014

[26] Tan, G. H., Goss, B. G., Thorpe, P. J. & Williams, R. P. CT-based classification of long spinal allograft fusion. *Eur. Spine J.* **16**, 1875–1881. 10.1007/s00586-007-0376-0 (2007). 17497188 10.1007/s00586-007-0376-0 PMC2223338

[27] Bach, K. *et al.* Morphometric analysis of lumbar intervertebral disc height: An imaging study. *World Neurosurg.* **124**, e106–e118. 10.1016/j.wneu.2018.12.014 (2019). 10.1016/j.wneu.2018.12.014 30579030

[28] Spahn, G. *et al.* Measurement of intervertebral disc heights in the lumbar spine. Comparison of X-ray and magnetic resonance imaging, method of measurement and determination of inter-observer reliability. *Z. Orthop. Unfall*10.1055/a-1994-0879 (2023). 36758585 10.1055/a-1994-0879

[29] Tanaka, M. *et al.* Revision for cage migration after transforaminal/posterior lumbar interbody fusion: How to perform revision surgery?. *BMC Surg.* **22**, 172. 10.1186/s12893-022-01620-0 (2022). 35546229 10.1186/s12893-022-01620-0 PMC9092779

[30] Zhao, L., Zeng, J., Xie, T., Pu, X. & Lu, Y. Advances in research on Cage subsidence following lumbar interbody fusion. *Zhongguo Xiu Fu Chong Jian Wai Ke Za Zhi* **35**, 1063–1067. 10.7507/1002-1892.202104036 (2021). 34387439 10.7507/1002-1892.202104036 PMC8403988

[31] Choi, J. Y. & Sung, K. H. Subsidence after anterior lumbar interbody fusion using paired stand-alone rectangular cages. *Eur. Spine J.* **15**, 16–22. 10.1007/s00586-004-0817-y (2006). 15843972 10.1007/s00586-004-0817-y PMC3454564

[32] Wu, X. *et al.* Pedicle screw loosening: The value of radiological imagings and the identification of risk factors assessed by extraction torque during screw removal surgery. *J. Orthop. Surg. Res.* **14**, 6. 10.1186/s13018-018-1046-0 (2019). 30616575 10.1186/s13018-018-1046-0 PMC6322238

[33] Sun, D. *et al.* OLIF versus ALIF: Which is the better surgical approach for degenerative lumbar disease? A systematic review. *Eur. Spine J.* **32**, 689–699. 10.1007/s00586-022-07516-0 (2023). 36587140 10.1007/s00586-022-07516-0

[34] Phan, K., Maharaj, M., Assem, Y. & Mobbs, R. J. Review of early clinical results and complications associated with oblique lumbar interbody fusion (OLIF). *J. Clin. Neurosci.* **31**, 23–29. 10.1016/j.jocn.2016.02.030 (2016). 27349468 10.1016/j.jocn.2016.02.030

[35] Ohtori, S. *et al.* Mini-open anterior retroperitoneal lumbar interbody fusion: Oblique lateral interbody fusion for lumbar spinal degeneration disease. *Yonsei Med. J.* **56**, 1051–1059. 10.3349/ymj.2015.56.4.1051 (2015). 26069130 10.3349/ymj.2015.56.4.1051 PMC4479835

[36] Li, J. X., Phan, K. & Mobbs, R. Oblique lumbar interbody fusion: Technical aspects, operative outcomes, and complications. *World Neurosurg.* **98**, 113–123. 10.1016/j.wneu.2016.10.074 (2017). 27777161 10.1016/j.wneu.2016.10.074

[37] Mehren, C., Mayer, H. M., Zandanell, C., Siepe, C. J. & Korge, A. The oblique anterolateral approach to the lumbar spine provides access to the lumbar spine with few early complications. *Clin. Orthop. Relat. Res.* **474**, 2020–2027. 10.1007/s11999-016-4883-3 (2016). 27160744 10.1007/s11999-016-4883-3 PMC4965375

[38] Lin, G. X. *et al.* Clinical and radiologic outcomes of direct versus indirect decompression with lumbar interbody fusion: A matched-pair comparison analysis. *World Neurosurg.* **119**, e898–e909. 10.1016/j.wneu.2018.08.003 (2018). 30099187 10.1016/j.wneu.2018.08.003

[39] Hagan, M. J. *et al.* Pedicle screw placement using intraoperative computed tomography and computer-aided spinal navigation improves screw accuracy and avoids postoperative revisions: Single-center analysis of 1400 pedicle screws. *World Neurosurg.* **160**, e169–e179. 10.1016/j.wneu.2021.12.112 (2022). 34990843 10.1016/j.wneu.2021.12.112

[40] Xi, Z., Chou, D., Mummaneni, P. V. & Burch, S. The navigated oblique lumbar interbody fusion: Accuracy rate, effect on surgical time, and complications. *Neurospine* **17**, 260–267. 10.14245/ns.1938358.179 (2020). 32054142 10.14245/ns.1938358.179 PMC7136090

[41] Ohba, T., Ebata, S., Fujita, K., Sato, H. & Haro, H. Percutaneous pedicle screw placements: Accuracy and rates of cranial facet joint violation using conventional fluoroscopy compared with intraoperative three-dimensional computed tomography computer navigation. *Eur. Spine J.* **25**, 1775–1780. 10.1007/s00586-016-4489-1 (2016). 26957097 10.1007/s00586-016-4489-1

[42] Tian, N. F. & Xu, H. Z. Image-guided pedicle screw insertion accuracy: A meta-analysis. *Int. Orthop.* **33**, 895–903. 10.1007/s00264-009-0792-3 (2009). 19421752 10.1007/s00264-009-0792-3 PMC2899004

[43] Warren, S. I., Wadhwa, H., Koltsov, J. C. B., Michaud, J. B. & Cheng, I. One surgeon’s learning curve with single position lateral lumbar interbody fusion: Perioperative outcomes and complications. *J. Spine Surg.* **7**, 162–169. 10.21037/jss-21-13 (2021). 34296028 10.21037/jss-21-13 PMC8261560

[44] Kwee, M. M., Ho, Y. H. & Rozen, W. M. The prone position during surgery and its complications: A systematic review and evidence-based guidelines. *Int. Surg.* **100**, 292–303. 10.9738/intsurg-d-13-00256.1 (2015). 25692433 10.9738/intsurg-d-13-00256.1 PMC4337445

[45] DePasse, J. M., Palumbo, M. A., Haque, M., Eberson, C. P. & Daniels, A. H. Complications associated with prone positioning in elective spinal surgery. *World J. Orthop.* **6**, 351–359. 10.5312/wjo.v6.i3.351 (2015). 25893178 10.5312/wjo.v6.i3.351 PMC4390897

[46] Oliveira, L., Marchi, L., Coutinho, E. & Pimenta, L. A radiographic assessment of the ability of the extreme lateral interbody fusion procedure to indirectly decompress the neural elements. *Spine (Phila Pa 1976)* **35**, S331-337. 10.1097/BRS.0b013e3182022db0 (2010). 21160397 10.1097/BRS.0b013e3182022db0

[47] Oertel, M. F., Hobart, J., Stein, M., Schreiber, V. & Scharbrodt, W. Clinical and methodological precision of spinal navigation assisted by 3D intraoperative O-arm radiographic imaging: Technical note. *J. Neurosurg. Spine SPI* **14**, 532–536. 10.3171/2010.10.SPINE091032 (2011). 10.3171/2010.10.SPINE091032 21275555

[48] Park, P. Three-dimensional computed tomography-based spinal navigation in minimally invasive lateral lumbar interbody fusion: Feasibility, technique, and initial results. *Oper. Neurosurg.*10.1227/NEU.0000000000000726 (2015). 10.1227/NEU.0000000000000726 25812070

[49] Hiyama, A., Sakai, D., Sato, M. & Watanabe, M. The analysis of percutaneous pedicle screw technique with guide wire-less in lateral decubitus position following extreme lateral interbody fusion. *J. Orthop. Surg. Res.* **14**, 304. 10.1186/s13018-019-1354-z (2019). 31488181 10.1186/s13018-019-1354-z PMC6729011

[50] Chapman, T. M., Blizzard, D. J. & Brown, C. R. CT accuracy of percutaneous versus open pedicle screw techniques: A series of 1609 screws. *Eur. Spine J.* **25**, 1781–1786. 10.1007/s00586-015-4163-z (2016). 26219915 10.1007/s00586-015-4163-z

[51] Blizzard, D. J. & Thomas, J. A. MIS single-position lateral and oblique lateral lumbar interbody fusion and bilateral pedicle screw fixation: Feasibility and perioperative results. *Spine* **43**, 440–446. 10.1097/brs.0000000000002330 (2018). 28704331 10.1097/brs.0000000000002330

