# Supplementary material for: Single-position oblique lumbar interbody fusion with navigation: improved efficiency and screw accuracy compared to dual-position with fluoroscopy
Source: Sci Rep. 2024 Jul 23;14:16907. doi: 10.1038/s41598-024-67007-8 (PMC11266416; doi:10.1038/s41598-024-67007-8)
Supplement: Supplementary file 1 — Supplementary Figures. [file 41598_2024_67007_MOESM1_ESM.pptx]

## Slide 1
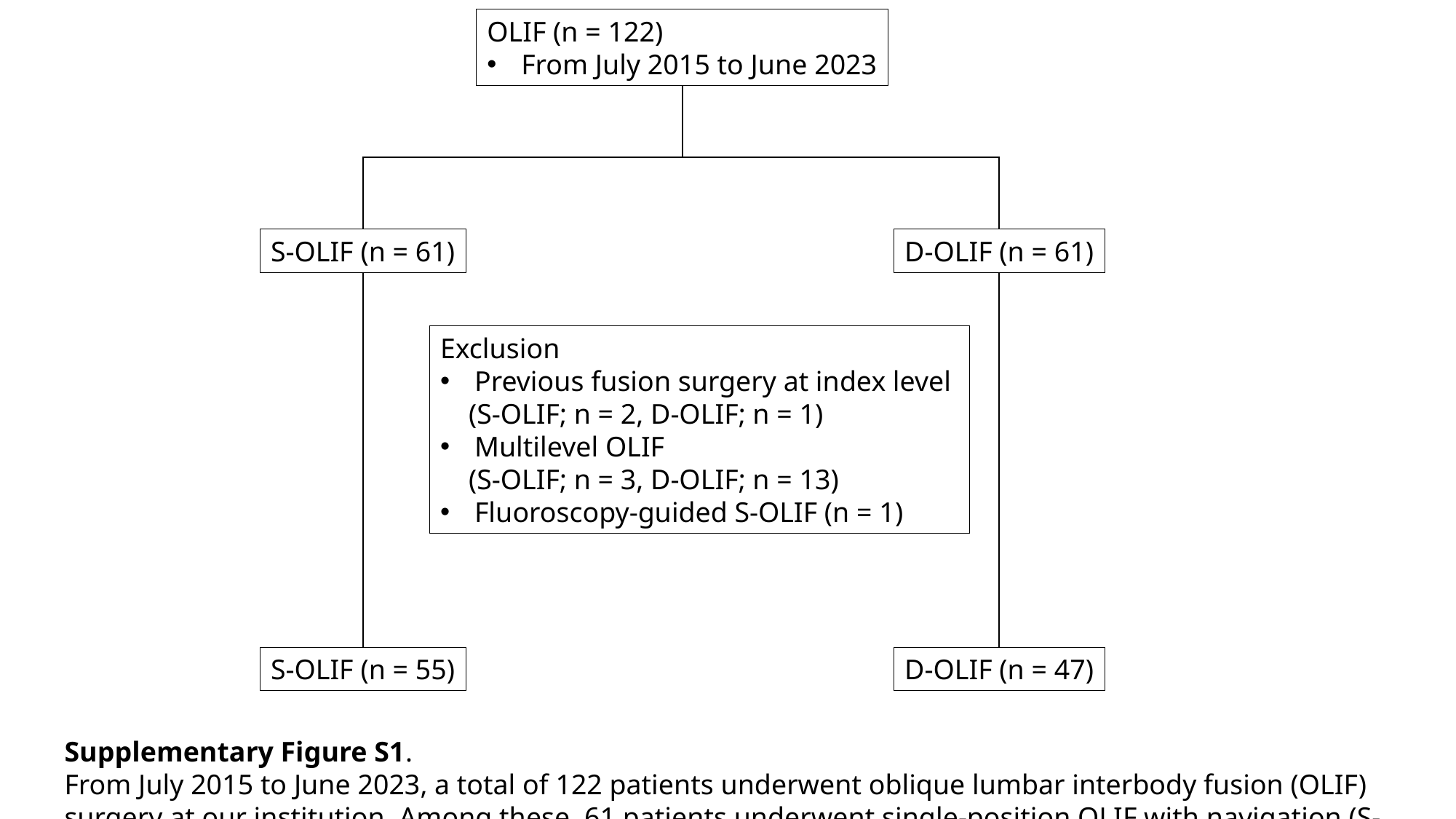

OLIF (n = 122)
From July 2015 to June 2023
S-OLIF (n = 61)
D-OLIF (n = 61)
Exclusion
Previous fusion surgery at index level
 (S-OLIF; n = 2, D-OLIF; n = 1)
Multilevel OLIF
 (S-OLIF; n = 3, D-OLIF; n = 13)
Fluoroscopy-guided S-OLIF (n = 1)
S-OLIF (n = 55)
D-OLIF (n = 47)
Supplementary Figure S1.
From July 2015 to June 2023, a total of 122 patients underwent oblique lumbar interbody fusion (OLIF) surgery at our institution. Among these, 61 patients underwent single-position OLIF with navigation (S-OLIF) and 61 patients underwent dual-position OLIF with fluoroscopy (D-OLIF). Patients who had previous fusion surgery at the index level (S-OLIF, n = 2; D-OLIF, n = 1) were excluded. Additionally, those who underwent multilevel OLIF (S-OLIF, n = 3; D-OLIF, n = 13) and one patient who underwent fluoroscopy-guided S-OLIF were excluded. Consequently, a total of 55 patients in the S-OLIF group and 47 patients in the D-OLIF group were included in the analysis for single-level OLIF.

## Slide 2
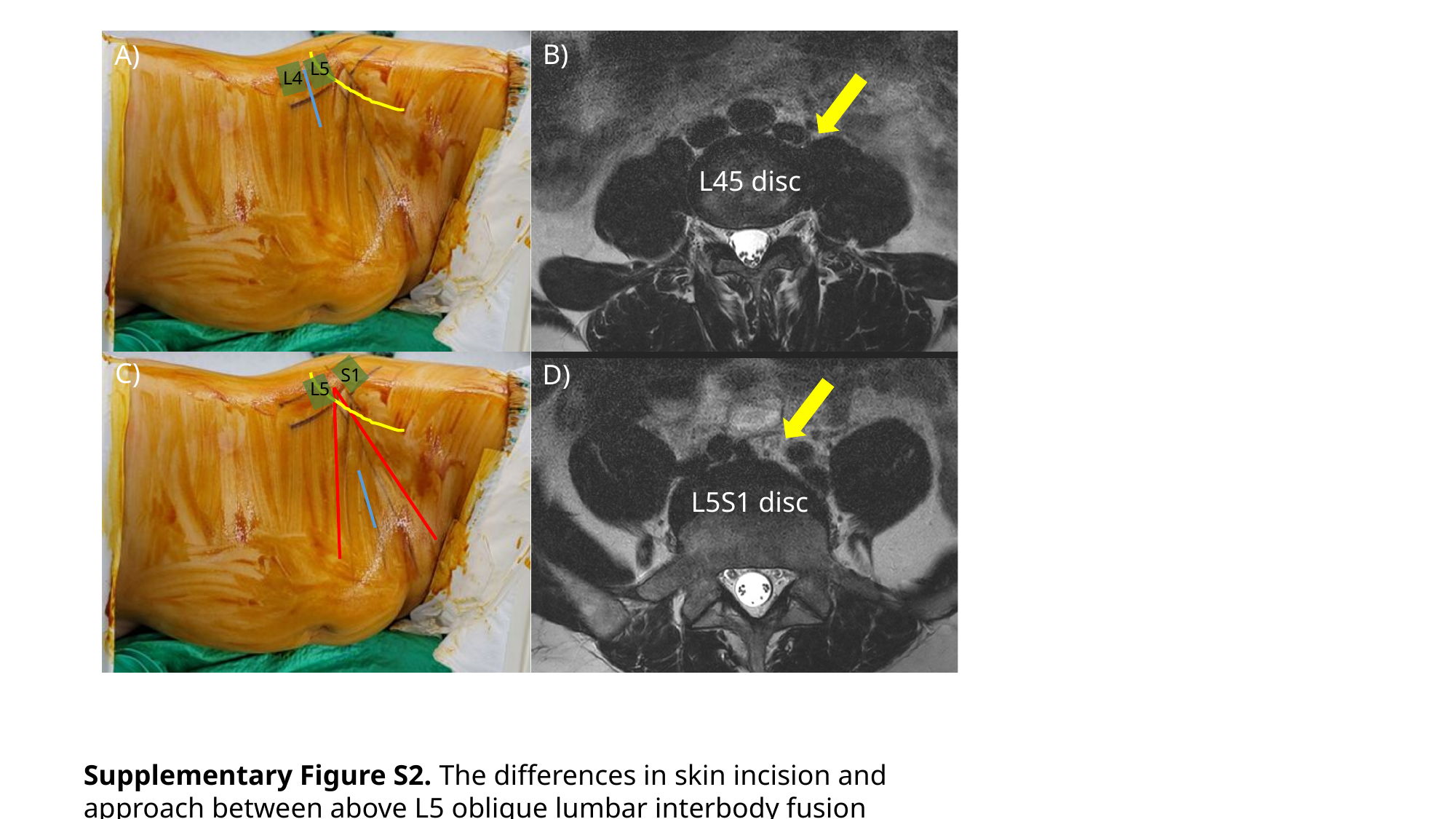

B)
A)
L5
L4
L45 disc
C)
D)
S1
L5
L5S1 disc
Supplementary Figure S2. The differences in skin incision and approach between above L5 oblique lumbar interbody fusion (OLIF) and L5-S1 OLIF.
A) For above L5 OLIF, a 6 cm skin incision (blue line) is made anteriorly and posteriorly from the anterior margin of the L4-5 disc, above the iliac crest (yellow line). B) The disc space is exposed by approaching between the left psoas muscle and the common iliac vessel, detaching the psoas muscle to reveal the mid-point of the index disc. C) For L5-S1 OLIF, a 6 cm skin incision (blue line) is made two finger-breadths anterior to the anterior superior iliac spine (ASIS), after drawing a vertical line perpendicular to the floor from the mid-point of the L5-S1 disc and a parallel line to the S1 endplate (red line). D) The disc space is exposed by approaching between the bilateral common iliac vessels.
