# Supplementary material for: Single-position oblique lumbar interbody fusion with navigation: improved efficiency and screw accuracy compared to dual-position with fluoroscopy
Source: Sci Rep. 2024 Jul 23;14:16907. doi: 10.1038/s41598-024-67007-8 (PMC11266416; doi:10.1038/s41598-024-67007-8)
Supplement: Supplementary file 2 — Supplementary Tables. [file 41598_2024_67007_MOESM2_ESM.docx]

Supplementary Table S1. Detail information of postoperative complications

| Complications | Number of patients | Symptoms | Treatment | Outcomes |
| --- | --- | --- | --- | --- |
| Ipsilateral transient psoas weakness | S-OLIF: 2  D-OLIF: 1 | Lt HF grade IV or IV+ | Supportive care | Lt HF grade V within mean PO 2.3 months |
| Peritoneal injury | S-OLIF: 1  D-OLIF: 1 |  | Primary repair | No further complications |
| Paralytic ileus | S-OLIF: 2  D-OLIF: 2 | Abdominal pain | Supportive care | Recovery within mean PO 2.8 days |
| Ipsilateral sympathetic chain symptoms | S-OLIF: 5  D-OLIF: 6 | Lt LE swelling, redness, loss of sweating | Supportive care | Recovery within mean PO 8.6 months^†^ |
| Irritation of ipsilateral genitofemoral nerve | S-OLIF: 9  D-OLIF: 1 | Lt anterior and/or medial aspect of thigh pain or sensory loss | Supportive care | Recovery within mean PO 9.5 months |

S-OLIF; single-position oblique lumbar interbody fusion with navigation, D-OLIF; dual-position oblique lumbar interbody fusion with fluoroscopy, Lt; left, HF; hip flexion, PO; postoperative, LE; lower extremity

^†^One patient in the D-OLIF group had persistent symptoms up to 6 months after surgery (last follow-up).

Supplementary Table S2. Clinical outcomes of multilevel S-OLIF

| No | Age/Sex | Diagnosis | Level | FU duration (months) | NRS-back / leg | ODI (%) | EQ-5D-5L | Complications | Secondary surgery |
| --- | --- | --- | --- | --- | --- | --- | --- | --- | --- |
| 1 | 73/F | L3-4: SPL, grade 1  L4-5: SPL, grade 1 | L3-5 | 15.8 | Preop: 10/10  Last FU: 2/2 | Preop: 57.8  Last FU: 22.2 | Preop: 0.29  Last FU: 0.795 |  |  |
| 2 | 76/M | L2-3: auto fusion  L3-4: FA  L4-5: FS | L3-5 | 17.2 | Preop: 7/8  Last FU: 6/8 | Preop: 68.9  Last FU: 71.1 | Preop: 0.331  Last FU: 0.493 |  |  |
| 3 | 75/F | L4-5: FS  L5-S1: SPL, grade 1 | L4-S1 | 13.4 | Preop: 8/8  Last FU: 6/4 | Preop: 53.3  Last FU: 40 | Preop: 0.485  Last FU: 0.646 | Transient Lt HF weakness | Cage re-insertion d/t L5-S1 cage migration |

S-OLIF; single position oblique lumbar interbody fusion with navigation, No; number, FU; follow-up, NRS; numerical rating scale, ODI; Oswestry Disabilityu Index, EQ-5D-5L; EuroQol 5-Dimension 5-Level, F; female, SPL; spondylolisthesis, Preop; preoperative, M; male, FA; facet arthropathy, FS; foraminal stenosis, Lt; left, HF; hip flexion, d/t; due to

Supplementary Table S3. Radiological outcomes of multilevel S-OLIF

| No | Segmental lordosis | Lumbar lordosis | Interbody fusion | Case position | Subsidence | Pedicle screw | Instrument failure |
| --- | --- | --- | --- | --- | --- | --- | --- |
| 1 | Preop: 10.7  Last FU: 18 | Preop: 43.5  Last FU: 58.5 | L3-4: fusion  L4-5: fusion | L3-4: anterior 1/3  L4-5: anterior 1/3 | L3-4: x  L4-5: x | L3: no cortical breach  L4: no cortical breach  L5: no cortical breach |  |
| 2 | Preop: 16.1^†^  Last FU: 15.8^†^ | Preop: 8.3  Last FU: 7.6 | L3-4: non-fusion  L4-5: non-fusion | L3-4: anterior 1/3  L4-5: anterior 1/3 | L3-4: x  L4-5: x | L3: no cortical breach  L4: no cortical breach  L5: no cortical breach | L3: both halo |
| 3^‡^ | Preop: 36.8  Last FU: 37.8 | Preop: 33.5  Last FU: 37.9 | L4-5: fusion  L5-S1: fusion | L4-5: middle 1/3  L5-S1: anterior 1/3 | L4-5: x  L5-S1: x | L4: no cortical breach  L5: no cortical breach  S1ala: no cortical breach  Iliac: no cortical breach | Iliac: both halo |

S-OLIF; single position oblique lumbar interbody fusion with navigation, No; number, Preop; preoperative, FU; follow-up

^†^The L2-5 segmental angle was measured due to L2-3 auto fusion.

^‡^ Outcomes after revision were presented.
